# Identification of Lipids and Cytokines in Plasma and Follicular Fluid before and after Follicle-Stimulating Hormone Stimulation as Potential Markers for Follicular Maturation in Cattle

**DOI:** 10.3390/ani13203289

**Published:** 2023-10-21

**Authors:** Alexandria P. Snider, Renata S. Gomes, Adam F. Summers, Sarah C. Tenley, Mohamed A. Abedal-Majed, Renee M. McFee, Jennifer R. Wood, John S. Davis, Andrea S. Cupp

**Affiliations:** 1 United States Department of Agriculture, Agricultural Research Service, U.S. Meat Animal Research Center, Clay Center, NE 68933, USA; alex.snider@usda.gov; 2 Department of Animal Science, University of Nebraska–Lincoln, 3940 Fair Street, Lincoln, NE 68583, USA; rspurigomes2@huskers.unl.edu (R.S.G.); jwood5@unl.edu (J.R.W.); 3 ReproLogix, 2031 Indian Road, Fort Scott, KS 66071, USA; asummers@rlteam.com; 4 Department of Animal Production, School of Agriculture, The University of Jordan, Amman 11942, Jordan; m.ayoub@ju.edu.jo; 5 School of Veterinary Medicine and Biomedical Sciences, University of Nebraska-Lincoln, Lincoln, NE 68583, USA; rmcfee3@unl.edu; 6 Olson Center for Women’s Health, University of Nebraska Medical Center, 983255 Nebraska Medical Center, Omaha, NE 68198, USA; jsdavis@unmc.edu; 7 VA Nebraska-Western Iowa Health Care System, Omaha, NE 68105, USA

**Keywords:** low-dose FSH, cow, cytokines, chemokines, lipids, estrus cycle, follicle maturation

## Abstract

**Simple Summary:**

The ovarian follicle contains an egg that must be matured appropriately to maintain regular reproductive cycles in cows and result in the ovulation of an egg capable of fertilization. Anovulation often occurs when there is poor follicle maturation. Lipids and cytokines in circulation and follicular fluid have been known to have an important role in follicular maturation. However, specific cytokine or lipid markers, or their role during stages of the cycle and when they are needed to provide optimal follicular maturation, have not been elucidated. The current study investigated the relationship between circulating and follicular fluid lipids and cytokines during an estrous cycle and after follicular stimulation with follicle-stimulating hormone. The identification of specific cytokines and lipids present at different stages of the reproductive cycle in circulation and the follicular fluid of the dominant follicle provides a springboard to study the mitigation of poor follicle maturation or anovulation. Future studies can also utilize the markers developed within this study to determine their role during follicular maturation.

**Abstract:**

The process of follicle maturation leading to ovulation is a key milestone in female fertility. It is known that circulating lipids and cytokines play a role in the follicle’s ability to go through follicular maturation and the ovulatory processes. However, the specific mechanisms are not well understood. We posit that dysregulation of granulosa cells influences the ovarian environment, which tries to adapt by changing released lipids and cytokines to achieve follicular maturation. Eleven non-lactating adult females underwent estrus synchronization with two injections of PGF2α 14 days apart. Daily blood samples were collected for 28 days to monitor steroid hormone production after the second injection. To understand the potential impacts of lipids and cytokines during ovulation, a low-dose FSH stimulation (FSHLow) was performed after resynchronization of cows, and daily blood samples were collected for 14 days to monitor steroid hormone production until ovariectomies. The lipidomic analysis demonstrated increased circulating diacylglycerides and triacylglycerides during the mid-luteal phase and after FSHLow treatment. Cholesteryl esters decreased in circulation but increased in follicular fluid (FF) after FSHLow. Increased circulating concentrations of TNFα and reduced CXCL9 were observed in response to FSHLow. Therefore, specific circulating lipids and cytokines may serve as markers of normal follicle maturation.

## 1. Introduction

Anovulation or irregular timing of ovulation is an issue in mammalian species [1,2,3]. The reproductive alterations that cause anovulation are not well understood. Therefore, identifying markers associated with ovulation would be beneficial for identifying new approaches to assist fertility or ovulation failure. Leading up to ovulation, pre-antral follicles undergo selection and development into a small antral follicle pool. Theca and granulosa cells within the antral follicles are involved in steroidogenesis, inflammation, and cellular development, leading to a dominant follicle being selected for ovulation [4,5]. Steroidogenesis is an important and influential component of folliculogenesis, as the theca and granulosa cells produce androgens and estrogens, respectively, which aid in the continuation of cellular development and other factors involved in the ovulation cascade [6].

Circulating lipids have important roles in cellular processes, such as steroidogenesis, cell proliferation, and metabolism [7,8,9]. Cholesterol utilized for steroid production is derived from the blood in the form of high- and low-density lipoprotein-derived cholesteryl esters [10]. Lipoproteins are internalized either through receptor-mediated endocytosis or selective cellular uptake, where cholesterol is sorted from lipoproteins in endosomes. Following intracellular processing, lipoprotein-derived cholesterol is trafficked to mitochondria for immediate progesterone biosynthesis or stored in lipid droplets as cholesteryl esters [10,11]. These hormones will either be released in circulation or further cleaved and released, targeting tissues to elicit their effect [12]. During a female’s reproductive cycle, steroid production changes based on the release of gonadotropins to support structures present in the ovary [13]. Lipid breakdown causes reactive oxygen species (ROS) to be released, increasing localized inflammation [14]. Increases in specific lipids, such as diacylglycerols (DG), triacylglycerides (TG), lysophosphatidylcholines (LysoPC), and phosphatidylcholines (PC), up-regulate cytokine production, leading to increased inflammatory response [15,16,17,18,19,20].

Superovulation is one of the assisted reproductive technologies (ART) commonly used in cattle to increase the number of dominant follicles present for ovulation. Follicle-stimulating hormone is used to promote the development of multiple follicles and ovulation [21]. Thus, our hypothesis was that the FSHLow protocol would increase pro-inflammatory lipids and cytokines, which, compared to times during the early and mid-luteal phase, would enable us to identify potential regulators (lipids, cytokine, and chemokines) of the follicular maturation process.

## 2. Material and Methods

### 2.1. Animals

Eleven non-lactating, composite [25% MARC III (1/4 Angus, 1/4 Hereford, 1/4 Pinzgauer, 1/4 Red Poll) and 75% Red Angus] beef cows from the beef physiology herd at the University of Nebraska Eastern Nebraska Research, Education, and Extension Center (ENREEC), were used in this study. All procedures were approved by the Animal Care and Use Committee at the University of Nebraska–Lincoln.

### 2.2. Estrous Synchronization and Stimulation

**Figure 1:**
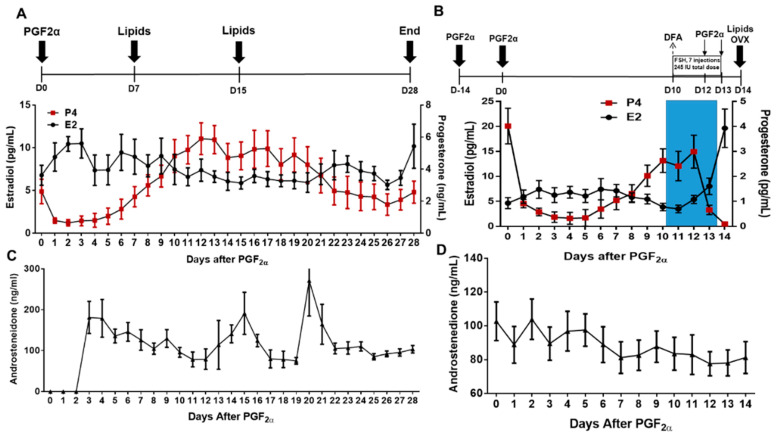
Estrus synchronization protocol for non-stimulated and stimulated cycles. (**A**) Estrous cycles of cows (n = 11) were synchronized with two injections of prostaglandin (PGF2α). After the second PGF2α injection, plasma was collected on day 7 after PGF2α- (Early Luteal; EL) and day 15 after PGF2α- (mid-luteal; ML); (**B**) in a subsequent trial, the same cows underwent a DFA on day 10 of the estrous cycle, then were stimulated with FSH (35 IU FSH every 12 h for 3.5 days plus PGF2α at the same time the last injection of FSH and 12 h after the last injection FSH), and FF and plasma samples were collected at ovariectomy (OVX; FSHLow = OVX). Blue shading denotes FSH stimulation. All plasma and follicular fluid samples were used for lipidomic and cytokine analysis. Average daily E2 and P4 (n = 11) are below each cycle. (**C**) Average daily concentrations of A4 (n = 11) for a non-stimulated cycle. (**D**) Average daily concentrations of A4 (n = 11) for a stimulated cycle.

Estrus was synchronized utilizing two injections of prostaglandin (PGF2α; 25 mg; i.m.; Lutalyse^®^, Zoetis Inc., Florham Park, NJ, USA) 14 days apart (Figure 1A). The time of the second injection was considered Day 0, and daily blood samples were collected to monitor estrogen, progesterone, and androstenedione concentrations throughout the following 28 days (Figure 1A,C). After day 28, cows were given two injections of PGF2α 14 days apart to resynchronize their cycles, and blood samples were collected daily for 14 days to monitor estrogen, progesterone, and androstenedione concentrations until the time of ovariectomies (Figure 1B). On day 10 of the second cycle, dominant follicle aspirations (DFA) were performed to collect follicular fluid for lipidomic and cytokine analysis. To ensure a new follicle wave would emerge, any follicles ≥ 5 mm in diameter were ablated. The standard dose of FSH (700 IU) can result in hyperstimulation in some individuals; thus, reduced FSH concentrations given (245 IU) [22] in the present study initiated the follicular maturation process while guarding against excessive stimulation of the ovary and allowing for investigation of factors surrounding ovulation, including small lipids, cytokines, and chemokines. As previously described by McFee et al. [22], the follicle-stimulating hormone (FSH; Follitropin-V; Bioniche Animal Health, Belleville, ON, Canada; each equivalent to 20 mg/mL NIH-FSH-P_1_) was administered intramuscular (i.m) twelve hours apart over 3.5 days, with a total of 7 injections administered. On days 12 and 13 of the cycle, cows received 2 i.m. injections of PGF2α 24 h apart. Ovariectomies were performed on all cows on day 14, approximately 24 h after the last PGF2α injection (Figure 1B). 

### 2.3. Blood Collections

Blood plasma was collected daily on all cows during both cycles to monitor steroid hormone profiles—androstenedione (A4), estradiol (E2), and progesterone (P4) —on day 7 after PGF2α (Early Luteal; EL) and day 15 after PGF2α (mid-luteal; ML) for lipidomic analysis, and at ovariectomy after FSH stimulation (FSH-Low) (Figure 1). Briefly, 7 mL of blood was collected via coccygeal venipuncture into glass vacutainer tubes containing K_3_ EDTA (Becton, Dickinson and Company, Franklin Lakes, NJ, USA) as previously described in our lab [23]. After collection, blood was placed on ice, transported back to the lab, and spun at 700× *g* at 4 °C for 30 min. Plasma was collected and stored at −20 °C in polypropylene tubes (Globe Scientific, Inc., Paramus, NJ, USA) until further analysis.

### 2.4. Ovariectomies

Ovaries were collected on day 14, 24 h after the last FSH injection utilizing a right flank incision using an aseptic technique [23,24]. Briefly, local anesthetic was administered utilizing an inverted L-block with 2% lidocaine, and an additional 30–60 mL of lidocaine was topically applied to the muscle layers and peritoneum prior to incision. Prior to the removal of the ovaries, a plastic umbilical clamp was applied to the ovarian pedicle to minimize hemorrhaging. Ovaries were transported back to the lab, and the largest follicle from each pair of ovaries was aspirated to isolate follicular fluid. The fluid was stored at −80 °C for further lipid and cytokine analysis. 

### 2.5. Steroid Hormone Assays

Daily E2 concentrations were determined in plasma utilizing a radioimmunoassay carried out as previously described [23,25]. Values from samples were corrected for an extraction efficiency of 89.25%. Intra- and inter-CVs were 8.17% and 14.31%, respectively. Daily plasma P4 concentrations were determined utilizing the ImmuChem™ Coated Tube (MP Diagnostics, Solon, OH, USA) without extraction [23]. The intra- and inter-assay coefficients of variation (CV) were 9.59% and 15.0%, respectively. Daily plasma A4 concentrations were determined using ImmunChem™ Double Antibody (MP Diagnostics, Solon, OH, USA), and A4 was extracted as previously described [23]. Sample values were corrected for an extraction efficiency of 78.81%. Intra and inter-assay CVs were 8.62% and 6.66%, respectively. 

### 2.6. Lipidomic Analysis

Plasma samples collected at EL, ML, and FSHLow, as well as follicular fluid collected at the time of dominant follicle aspirations and at the time of ovariectomies (Figure 1), were sent to the Bioanalysis and Omnis [ARC-BIO] Center of Colorado State University to examine the lipid content and profiling. Briefly, 50 µL of plasma was extracted with 450 μL of Methyl tert-butyl ether (MTBE):Methanol (2:1, v:v). The sample was vortexed centrifuged, and the supernatant was transferred to a new 2.0 mL autosampler vial. The solvent was evaporated under a stream of nitrogen and resuspended in 100 μL of toluene:methanol (3:2, v:v). Then, the UPLC–MS Analysis (CSH PhenylHexyl method) was used, with 5 μL of extract injected twice (n = 2 replicates) into a Waters Acquity UPLC system in discrete, randomized blocks and separated using a Waters Acquity UPLC CSH Phenyl Hexyl column (1.7 μM, 1.0 × 100 mm), using a gradient from solvent A (water, 0.1% formic acid) to solvent B (Acetonitrile, 0.1% formic acid, 2 mM Ammonium Hydroxide). Injections were made in 100% A, held at 100% A for 1 min, ramped to 98% B over 12 min, held at 98% B for 3 min, and then returned to starting conditions over 0.05 min and allowed to re-equilibrate for 3.95 min, with a 200 μL/min constant flow rate. The column and samples were held at 65 °C and 6 °C, respectively. The column eluent was infused into a Waters Xevo G2 Q-TOF-MS with an electrospray source in positive mode, scanning 50–2000 m/z at 0.2 s per scan, alternating between MS (6 V collision energy) and MSE mode (15–30 V ramp). Calibration was performed using sodium iodide with 1 ppm mass accuracy. The capillary voltage was held at 2200 V, the source temperature was at 150 °C, and the nitrogen desolvation temp was at 350 °C with a flow rate of 800 L/h [26]. 

### 2.7. Cytokine Analysis

Plasma was collected at EL, ML, and FSHLow time points. Follicular fluid was collected at the time of DFA and FSHLow time points. Both plasma and FF were used to determine cytokine concentration with commercially available antibody arrays (Quantibody Cytokine Q3 and Q1 Arrays, RayBiotech, GA, USA. There were 20 different cytokines and chemokines in the pro- or anti-inflammatory pathway arrays including: ANG1 (Angiopoietin 1), CD40 Ligand, CCL4 (Chemokine (C-C motif) Ligand 4, also known as Macrophage inflammatory protein-1β (MIP-1β)), CCL5 (Chemokine (C-C motif) Ligand 5, also known as RANTES –Regulated on Activation, Normal T Cell Expressed and Secreted), CXCL9 (Chemokine (C-X-C Motif) Ligand 9 (also known as MIG- Macrophage Interferon gamma), CXCL10 (C-X-C Motif Chemokine Ligand 10, also known as Interferon–Inducible Cytokine IP-10), Decorin, IFNβ (Interferon Beta 1), IFNγ (Interferon Gamma), IL18 (Interleukin-18), LIF (Leukemia inhibitory factory), IL13 (Interleukin 13), IL21(Interleukin 21), IL36RA (Interleukin 36 Receptor Antagonist, also known as IL1F5—Interleukin 1 family member 5), TNFα (Tumor Necrosis Factor alpha), INFαA (Interferon alpha A), IL-10 (Interleukin 10), and IL17αA (Interleukin 17 alpha, also known as Cytotoxic T-Lymphocyte-Associated Protein 8) and have been validated for bovine samples [27,28]. Blood and follicular fluid were assayed using a 1 to 20 and 1 to 100 dilution, respectively. Array slides were shipped to the company for data extraction and retrieval. Data was received from RayBiotech and analyzed with the Q-Analyzer^®^ software (RayBiotech, Peachtree Corners GA, USA).

### 2.8. Statistical Analysis

Plasma hormone concentrations from non-stimulated and FSHLow time points were analyzed using the MIXED procedure of SAS with day as a fixed effect in a repeated measures model. For lipids analysis, for each sample, raw data files were converted to .cdf format, and a matrix of molecular features as defined by retention time and mass (m/z) was generated using XCMS software version 3.22.0 in R [29] for feature detection and alignment. Raw peak areas were normalized to the total ion signal in R, outlier injections were detected based on the total signal and PC1 of principle component analysis, and the mean area of the chromatographic peak was calculated among replicate injections (n = 2). Features were grouped based on a novel clustering tool, RAMClustR [30], which groups features into spectra based on co-elution and covariance across the full dataset, whereby spectra are used to determine the identity of observed compounds in the experiment. Compounds were annotated based on spectral matching to in-house, NISTv14, and Metlin metabolite databases. The peak areas for each feature in a spectrum were condensed via the weighted mean of all features in a spectrum into a single value for each compound. Analysis of variance was conducted on each compound using the AOV function in R, and *p*-values were adjusted for false positives using the Benjamini–Hochberg method in the p.adjust function in R [31]. Principal Component Analysis (PCA) was conducted on mean-centered and Pareto variance-scaled data using the pcaMethods package in R. 

Considering the high dimensionality of the dataset, three different machine-learning algorithms (Random Forest, Classification Tree, and the F selector) were used to select the most important discriminatory compounds. After examining the output of all three algorithms, the top ten compounds were selected to validate the predictions and determine the magnitude of differences between treatments. The choice of ten compounds was based on the mean decrease gene value depicted by Random Forest in addition to preventing an increased false positive discovery rate in discriminating between groups. Ultimately, a non-parametric *T*-test using a false discovery rate *p*-value of 0.05 was used to assess the magnitude of the effect of each selected compound. 

Plasma and follicular fluid lipid compounds that were annotated with known function were analyzed using the MIXED procedure in SAS with time as a fixed effect in a repeated measures model. Cytokine array analysis for plasma and follicular fluid between non-stimulated and FSHLow time points used the MIXED procedure of SAS with time as a fixed effect in a repeated measures model. Correlations between plasma steroid hormone (E2, P4, or A4) and plasma and follicular fluid lipid or cytokine profiles were analyzed utilizing the PROC CORR procedure in SAS. Significance was set at *p* < 0.05, with tendencies set at *p* = 0.05–0.1. 

## 3. Results

### 3.1. Steroid Profile before and after FSH Stimulation

**Figure 2:**
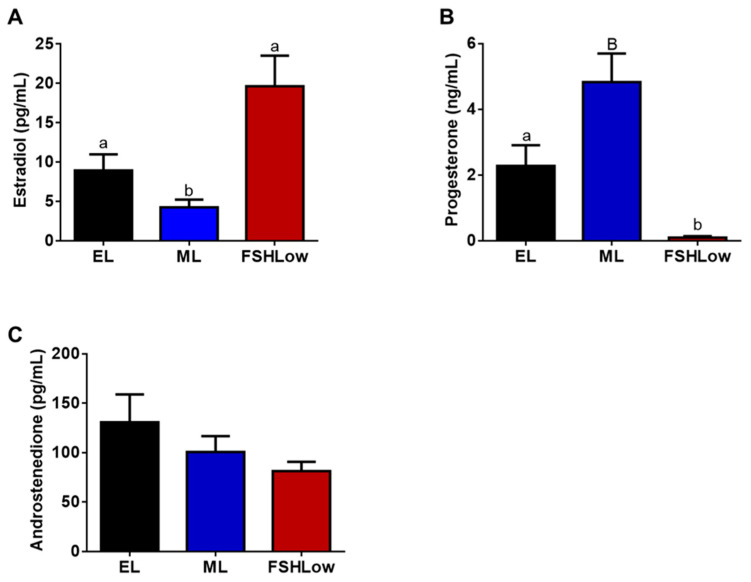
Plasma estradiol (**A**), progesterone (**B**), and androstenedione (**C**) concentrations at EL, ML, and in response to FSHLow. Analysis of steroid hormones was performed as described in the Methods. Data presented as means ± SEM, n = 11; differences in large letters *p* = 0.05–0.1 and small letters *p* < 0.05.

Plasma concentrations of E2, P4, and A4 concentrations were analyzed during a period of the trial that corresponded with lipidomic and cytokine analysis (Figure 2). Plasma estradiol concentrations at ML were decreased (*p* < 0.05) compared to EL and higher after FSH stimulation (*p* < 0.05; Figure 2A). Plasma progesterone had a tendency (*p* = 0.06) to be increased at ML compared to EL and significantly reduced (*p* < 0.01) in FSHLow compared to both EL and ML (Figure 2B). No differences were observed in plasma A4 concentrations when compared across all time points (Figure 2C). 

### 3.2. Plasma Lipidomic Profiles before and after FSH Stimulation

**Figure 3:**
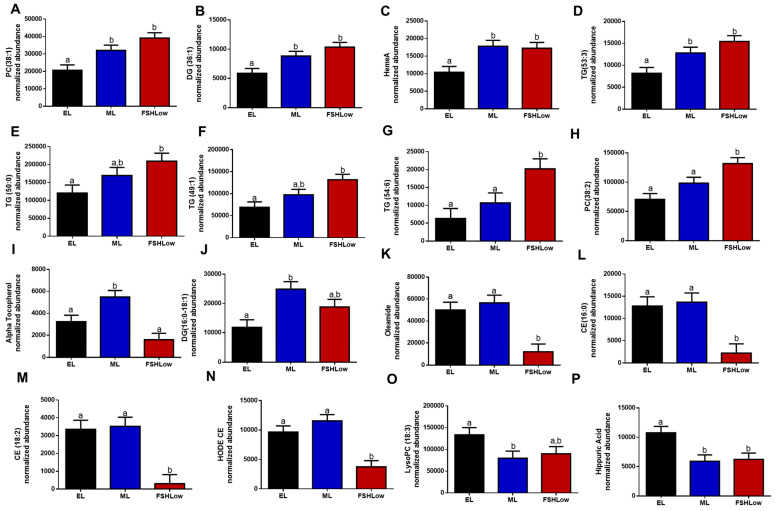
Selected circulating plasma lipids (**A**) PC (38:1), (**B**) DG (36:1), (**C**) HemeA, (**D**) TG (53:3), (**E**) Alpha Tocopherol, (**F**) TG (54:1), (**G**) TG (54:6), (**H**) PC (38:2), (**I**) TG (50:0), (**J**) DG (16:0–18:1), (**K**) Oleamide, (**L**) CE (16:0), (**M**) CE (18:2), (**N**) HODE CE, (**O**) LysoPC (18:3), (**P**) Hippuric Acid at EL, ML, and in response to FSHLow. Selected lipid profiles in plasma at EL, ML, and FSHLow. Analysis of normalized abundance of lipids was performed as described in the Methods. Data presented as means ± SEM, n = 11; differences in small letters *p* < 0.05.

The HPLC Mass Spec lipidomic analysis identified 863 lipid compounds. Retention times were determined for each compound, resulting in the identification of 155 annotated compounds. All lipid compounds in plasma and follicular fluid are presented in Supplementary Tables S1 and S2. Annotated lipids in plasma that were significantly different based on ANOVA were organized based on their reported role in inflammatory responses (Figure 3). Circulating phosphatidylcholine (PC) (38:1), an anti-inflammatory lipid, Diacylglycerol (DG) (36:1), HemeA and TG (53:3), associated with the pro-inflammatory response, were increased in blood plasma at mid-luteal (ML) and FSHLow compared to the early luteal phase (EL) (Figure 3A–D). Plasma TG (50:0), TG (49:1), TG (50:0), and PC (38:2) were increased at the FSHLow time point compared to EL (Figure 3E–H). In contrast, alpha-tocopherol (Vitamin E), an anti-inflammatory lipid, was increased during the ML when compared to EL and FSHLow (Figure 3I), similar to DG (16:0–18:1), which was increased in blood plasma at ML compared to EL but not different at FSHLow (Figure 3J). Oleamide (cis-9-octadecenamide), cholesteryl ester (CE) (16:0), CE (18:2), and HODE CE were reduced in circulation at FSHLow compared to EL and ML (Figure 3K–N). Circulating concentrations of pro-inflammatory Lyso-PC (18:3) were reduced at ML only compared to EL (Figure 3O). Hippuric acid (benzoylglycine), which is both a pro- and anti-inflammatory lipid, was reduced at ML and FSHLow when compared to EL (Figure 3P).

### 3.3. Follicular Fluid Lipidomic Profiles before and after FSH Stimulation

**Figure 4:**
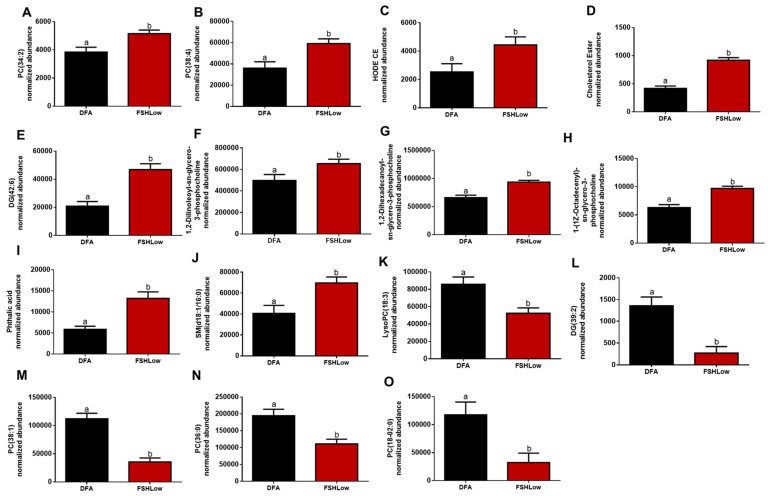
Selected follicular fluid lipids (**A**) PC (34:2), (**B**) PC (38:4), (**C**) HODE CE, (**D**) Cholesterol Ester, (**E**) PC (38:4), (**F**) 1,2-Dilinoleoyl-sn-glycero-3-phosphocholine, (**G**) 1,2-Dihexadecanoyl-sn-glycero-3-phosphocholine, (**H**) 1-(1Z-Octadecenyl)-sn-glycero-3-phosphocholine, (**I**) Phtalic acid, (**J**) SM(d18:0/16:0), (**K**) LysoPC (18:3), (**L**) DG (39:2), (**M**) PC (38:1), (**N**) PC (36:0), and (**O**) PC (18:0–02:0) at DFA and FSHLow. Selected lipid profiles in follicular fluid at DFA and FSHLow. Analysis of normalized abundance of lipids was performed as described in the Methods. Data presented as means ± SEM, n = 11; differences in small letters *p* < 0.05.

Follicular fluid was collected at DFA, and ovex following FSH (FSHLow) had altered lipid profiles, indicating a potential shift in the inflammatory response (Figure 4). There was an increase in FF PC (34:2) and PC (38:4), which are involved with the anti-inflammatory response (Figure 4A,B) from DFA to FSHLow. At FSHLow, HODE cholesteryl ester, cholesteryl ester, DG (42:6), and three sn-glycero-3-PC groups were increased compared to DFA (Figure 4C–H). Cholesteryl esters play a role in both the pro- and anti-inflammatory response and are involved with steroidogenesis. Lipids, phthalic acid, and SM (d18:1/14:0), involved with the pro-inflammatory response, were increased in FF at FSHLow (Figure 4I,J) compared to DFA. A reduction was observed in pro-inflammatory lipids lysophosphatidylcholine (Lyso-PC) (18:3) and DG (39:2) at FSHLow (Figure 4 K,L) compared to DFA. Follicular fluid PC (38:1), PC (36:0), and PC (18:0–02:0), which are involved with the anti-inflammatory response, were reduced at FSHLow (Figure 4M–O). For lipids involved with the pro-inflammatory response, there was a reduction in lysophosphatidylcholine (Lyso-PC) (18:3) and DG (39:2) (Figure 4J,K). In contrast, there was an increase of DG (42:6), phthalic acid, and SM (d18:1/16:0) in the follicular fluid at FSHLow (Figure 4L–N).

### 3.4. Plasma and Follicular Fluid Correlations of Lipids before and after FSH Stimulation

Circulating E2, P4, and A4 were correlated with lipids in circulation during the EL, ML, and FSHLow. A correlation was observed (Table 1) between the steroid hormones and follicular fluid lipids from DFA and FSHLow time points (Table 1). No significant correlations were observed with plasma steroid hormones and circulating lipids during the ML phase. At FSHLow, circulating E2 was negatively correlated with TG (54:6), TG (58:6), and HemeA (Table 1). Lipids positively correlated with A4 were detected during EL and were cholesteryl-11-hydroperoxy-eicosateraenoate, DG (38:0), TG (49:1), TG (50:0), TG (54:6), and TG (58:6) in circulation (Table 1). During EL, plasma P4 was positively correlated with PC (38:2), 3-Deoxyvitamin, Oleamide, sn-glycero-3-phosphoserine, CE (16:0), C.E., HODE, DG (16:0–18:0), DG (42:6), and TG (52:0) (Table 1). 

In the follicular fluid, circulating E2 was negatively correlated with 1,2-dilinoleoyl-sn-glycero-3-phosphocholine and Lyso-PC (20:4) at the time of DFA, while phthalic acid and HemeA were negatively correlated at FSHLow (Table 2). When follicular fluid was collected at DFA, circulating A4 was negatively correlated with PC (34:2) and PC (36:0), while 1-oleylo-2-hydroxy-sn-glycero-3-phosphocholine was positively correlated (Table 2). When the FSH was administered, a negative correlation was observed in plasma A4 with sodium glycochenodeoxycholate, and a positive correlation was observed with DG (42:6) (Table 2). Circulating P4 was positively correlated with phthalic acid in follicular fluid at the time of DFA and 1,2-dilinoleyol-sn-glycero-3-phosphocholine at FSHLow (Table 2). 

### 3.5. Plasma and Follicular Fluid Cytokine Profiles before and after FSH Stimulation

**Figure 5:**
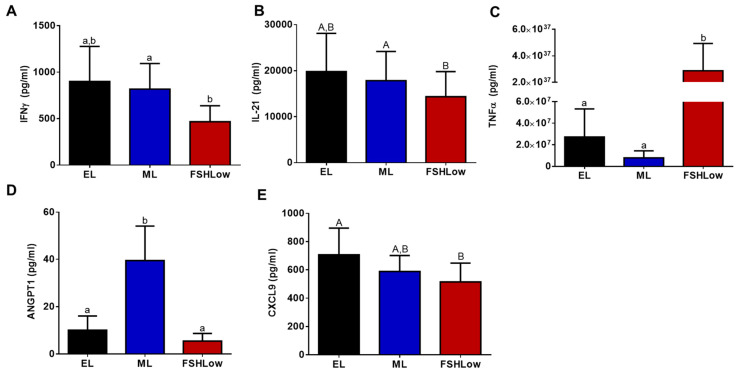
Circulating plasma production of cytokines during different times of the estrous cycle. Circulating IFNγ (**A**), IL-21 (**B**), TNFα (**C**), ANGPT1 (**D**), and CXCL9 (**E**). Data presented as means ± SEM, n = 11, with differences in large letters *p* = 0.05–0.1 and small letters *p* < 0.05.

**Figure 6:**
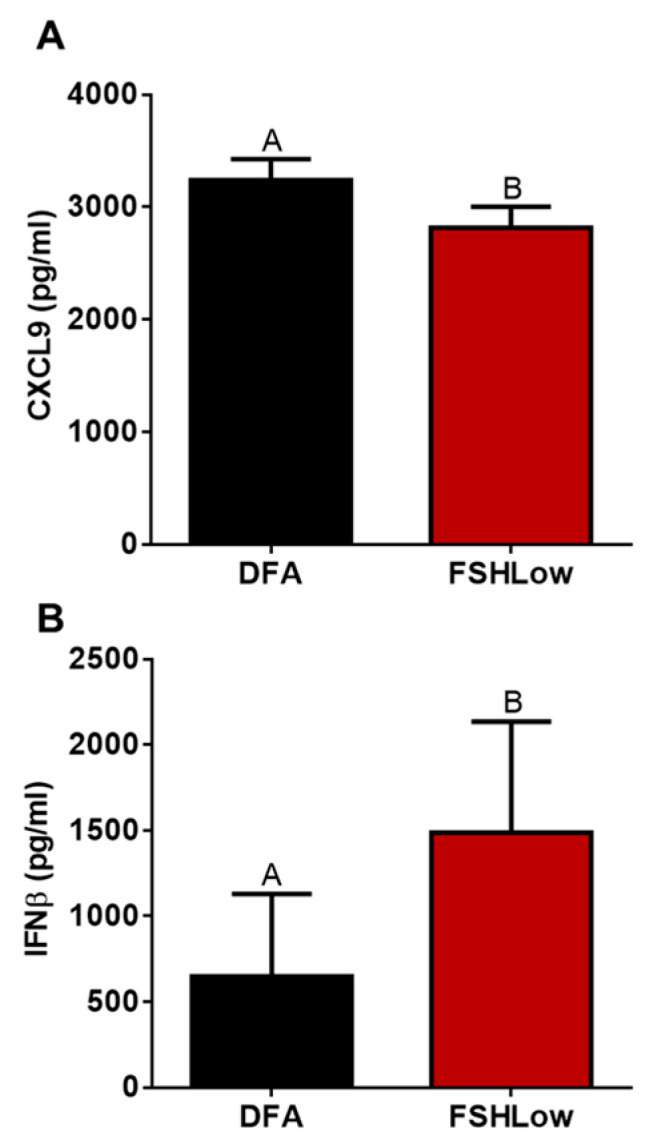
Follicular fluid cytokine profiles between DFA (EL) and FSHLow samples. Follicular fluid CXCL9 (**A**) and IFNβ (**B**). Data presented as means ± SEM, n = 11, with differences in large letters *p* = 0.05–0.1.

Cytokine concentrations were measured both in plasma and follicular fluid at the same time points and in animals as the lipidomic analysis. Based on the cytokine analysis, there were differences in five pro-inflammatory cytokines in circulation and two in follicular fluid (Figure 5A–E and Figure 6A,B). In plasma, there was a reduction (*p* < 0.05) in interferon-gamma (IFNγ) and a tendency (*p* = 0.07) for interleukin 21 (IL-21) to be reduced at FSHLow compared to EL (Figure 5A,B). At FSHLow, TNFα was significantly increased (*p* < 0.05) compared to EL and ML of the non-stimulated cycle (Figure 5C). Angiopoietin 1 (ANGPT1) was significantly increased (*p* < 0.05) at ML compared to EL in circulation (Figure 5D). C-X-C Motif Chemokine Ligand 9 (CXCL9) tended to be reduced at FSHLow compared to EL (Figure 5E).

In the follicular fluid at FSHLow, CXCL9 tended (*p* = 0.07) to be decreased compared to the non-stimulated cycle (Figure 6A). Interferon beta (IFNβ) had a tendency (*p* = 0.09) to be increased at FSHLow compared to the non-stimulated cycle (Figure 6B). 

### 3.6. Plasma and Follicular Fluid Correlations of Cytokines and Circulating Steroids before and after FSH Stimulation

Circulating steroid hormones E2, P4, and A4 were correlated with cytokines in circulation at EL, ML, and FSHLow. Circulating E2 was positively correlated with plasma IL-10, IL-18, IFNα, Decorin, and RANTES at FSHLow (Table 3). In plasma during EL, A4 was negatively correlated with RANTES, while A4 was positively correlated with IL-1β, IL-1F5, and TNFα (Table 3). During the ML phase, plasma A4 was positively correlated with IL-13, IL-21, MIG, IL-1β, and IL-17A (Table 3). At FSHLow, circulating A4 was positively correlated with IL-21 and MIG (Table 3). Moreover, plasma P4 was positively correlated with CD40L at the EL phase (Table 3). 

Follicular fluid cytokines at DFA and FSHLow were correlated with plasma steroid hormones for a non-stimulated cycle. Plasma E2 was negatively correlated with follicular fluid MIG at DFA (Table 4). Follicular fluid RANTES was positively correlated with circulating P4 at DFA (Table 4). At FSHLow, follicular fluid ANGPT1 was positively correlated with plasma progesterone (Table 4). No correlations were observed with plasma A4 and follicular fluid cytokines at DFA or FSHLow. 

## 4. Discussion

In the current study, we demonstrated alterations in lipid and cytokine profiles during the early and mid-luteal phases and after FSH stimulation. These were also correlated with circulating steroid hormone concentrations. Changes in lipids and cytokines during these specific phases might be an indicator of folliculogenesis and potentially peri- or pre-ovulatory status in females. 

### 4.1. Lipid and Cytokine Profiles during Early to Mid-Luteal Phase

The early to mid-luteal transitionary phase occurs during the period of potential early embryonic development to the point of maternal recognition of pregnancy [32]. During the luteal phase, circulatory lipids fluctuate, allowing increased numbers of lipid droplets within tissues to aid in inflammatory responses and energy storage [33]. In the current study, as the cows transition from the EL to the ML phase, circulatory lipids such as TGs, PCs, and DGs increase; this may allow for increased numbers of lipid droplets to be present for increased steroidogenesis of the developing CL [11] and develop within tissues to aid in inflammatory responses and energy storage [33]. Diacylglycerides are involved with signal transduction and regulation of PKC movement within the cell, impacting hormone and cytokine production [34,35]. Triacylglycerides have an important role in providing energy stores in the form of lipid droplets within tissues; specifically, lipid droplets in the CL provide a substrate for cellular metabolism and steroid synthesis [11,36]. HemeA was increased in circulation in the ML phase; this lipid is involved with cellular growth and survival through multiple pathways [37] and is primarily critical for aerobic respiration and generation of ATP and protecting against inflammation/oxidative stress (reviewed in [38]). A combination of these lipids has been shown to regulate the inflammatory pathway by stimulating the production of cytokines and chemokines that elicit an inflammatory response [37,39]. Angiopoietin-1 (ANGPT1) was increased in circulation during the ML phase of the reproductive cycle, as was Alpha Tocopherol (Vitamin E). Increased concentrations of ANGPT1 can lead to reduced inflammation, apoptosis, and fibrosis within tissues [40,41]. Reduction in inflammation and apoptosis is important for reproductive tissues since apoptosis results in a reduction in cell proliferation, follicular atresia, and CL regression [42]. Another important role of ANGPT1 is stabilizing vasculature within cells and tissues [41]. Increased vasculature is critical during the transition period from the EL to ML phase, as the CL develops and requires increased blood flow to allow for greater secretions of progesterone. Elevated progesterone is necessary for increasing uterine receptivity to a potential conceptus. As increased steroidogenesis occurs during ML, the elevated Alpha Tocopherol may act as an antioxidant to scavenge free radicals and reduce oxidative stress. In studies with women, Alpha Tocopherol increased in blood serum at times of increased steroidogenesis [43]. Steroids, lipids, and cytokines are produced and target specific cell types to enhance progesterone production and follicular development from the EL to ML phase of the reproductive cycle. Steroid hormones play a role in cytokine regulation and ovarian androstenedione production, increasing pro-inflammatory cytokine concentrations from the ovarian cortex [44]. 

In the current study, increased circulating androstenedione concentrations elevated circulating pro-inflammatory cytokines during the ML phase of the estrous cycle. After ovulation occurs, there is a rise in progesterone, leading up to a plateau during the ML phase [45]. The increase in progesterone concentration is due to an active CL present in the ovary during the ML phase of the estrous cycle [46]. Increased concentrations of circulating progesterone were observed to be negatively correlated with cytokines involved with the pro-inflammatory response. The mechanism of how steroid hormones, specifically androstenedione and progesterone, regulate cytokines is not fully understood and requires further investigation.

### 4.2. Lipid and Cytokine Profiles after FSHLow

Ovulation is classified as an inflammatory event culminating in the rupture of the dominant follicle, releasing a maturing oocyte for potential fertilization [6]. To understand and identify markers associated with folliculogenesis and selection of a dominant follicle, an FSHLow protocol was used to increase the number of dominant follicles developing at one time [22]. As demonstrated by Gwynne and Strauss [47], there is an increase in the inflammatory pathway with several potential lipids involved during folliculogenesis and dominant follicle selection. In the current study, after FSHLow, DGs and TGs increased in circulation. DGs are intermediates of extracellular lipid metabolism and are generated during TGs hydrolysis. DGs are also generated through hydrolysis of lipoprotein-associated TGs via the action of the enzyme’s lipoprotein lipase and hepatic lipase [48]. These lipids are involved with the pro-inflammatory response; specifically, DGs are important for regulating the PKC pathway [49,50,51,52]. The PKC pathway regulates protein hormone synthesis, such as FSH and LH production within the anterior pituitary [53]. The DGs are also involved with PKC pathway regulation, providing a substrate to enhance the secondary messenger system [54]. The combination of these lipids increases substrate availability for hormone production, aiding in increased follicular development during the FSHLow stimulation protocol. Interestingly, blood plasma cholesterol esters and Oleamide were decreased after FSHLow. The reduction in cholesterol esters may be due to increased cholesterol esters being taken up by the ovary to be utilized in steroidogenesis. Cholesterol esters can be scavenged from plasma lipoproteins in a selective cholesteryl ester uptake process [55]. Thus, FSH stimulation may mobilize cholesterol after FSHLow to increase estrogen production prior to ovulation (reviewed in [56]). Oleamide is a derivative of oleic acid and can act as a signaling molecule. It has been shown to interact with voltage-gated Na channels, GABA, and other receptors. One of the primary effects of its actions is vasodilation. Thus, there is potential that Oleamide may have been utilized before or during FSH stimulation and was depleted after FSHLow in the current study [57].

The development of antral follicles causes granulosa and theca cells to undergo cellular restructuring in preparation for potential ovulation. These cells require energy and substrates for cellular divisions, differentiation, or apoptosis, depending on the signals obtained from the surrounding tissues [58,59]. HemeA is required for cellular energy and oxygen utilization while regulating cellular inflammation [37,60]. This specific lipid was shown to increase in circulation after the FSHLow stimulation protocol, which can contribute to increased cellular inflammatory pathways due to the increased presence of antral follicles. Since the pro-inflammatory response is an important aspect of the ovulatory cascade, increased circulating concentrations of these lipids might be an indicator of the ovulatory cascade being initiated. We have shown that lipids were correlated with steroids in circulation after FSHLow, specifically E2, which was negatively correlated with TGs and HemeA. This observation could be a result of lipids being sequestered and utilized in reproductive tissues, leading to reduced concentrations in circulation. 

Increased inflammation can trigger changes in circulating cytokine profiles that target specific tissues, aiding in a localized inflammatory response [61]. During the FSHLow treatment, multiple antral follicles develop to a dominant state, causing an increase in the pro-inflammatory response observed in circulation [6]. We have shown that one major cytokine involved with the inflammatory response, TNFα, was significantly increased after FSHLow when compared to EL and ML. Increased circulation of TNFα activates monocytes to M1 macrophages, aiding in the pro-inflammatory response within the dominant follicle. Reduction in circulating interferon-gamma (IFNγ), IL-21, and ANG-1 were observed after FSHLow compared to ML, while CXCL9 tended to be reduced after FSHLow compared to EL. IFNγ is classified as a pro-inflammatory cytokine, but it has anti-inflammatory effects that inhibit pro-inflammatory interleukins (ILs) and increase apoptosis of leukocytes within the body [62]. The combination of lipids and cytokines within circulation during this phase can be related to an increased number of antral follicles developing to the dominant stage. Estradiol peaked after FSHLow and was positively correlated with cytokines involved in the anti-inflammatory response; this could be a result of preparing the anti-inflammatory response post-ovulation and allowing for cellular remodeling and repair within the follicle. 

### 4.3. Follicular Fluid Lipid and Cytokine Profiles after FSHLow

The follicular fluid contains proteins, hormones, lipids, and cytokines that aid the dominant follicle in preparation for potential ovulation [63,64,65]. Follicular fluid cholesterol was increased after FSHLow, which may indicate increased lipoprotein uptake. Our FSHLow protocol also increased the number of antral follicles initiating the ovulation cascade. While preparing for ovulation, specific lipids, such as sphingomyelin, present in the FF, have been shown to be involved with cellular apoptosis and inflammation [66,67]. Sphingomyelin is a component of the plasma membrane of cells and has also been shown to be a regulator of cellular proliferation and differentiation [68]. Increased production can be a result of greater pro-inflammatory cytokine from a localized inflammatory response [69]. In a study by Abdulrahman Alrabiah et al., bovine FF from peri-ovulatory follicles had an increase in sphingomyelin nearing ovulation, which is supportive of the results shown in this study [70]. Phosphatidylcholine is another important component of the plasma membrane and has been shown to be increased in FF after ovarian stimulation [71]. One specific phosphatidylcholine, PC (34:2), was shown to be a potential marker for embryo quality and embryonic genome activation [65]. In this study, PC (34:2) was increased in abundance at the FSHLow timepoint, which is close to ovulation. Recent studies have shown an abundance of lipids in the FF of small antral follicles that can be indicative of follicular maturation success and possibly oocyte quality [72,73]. This study supports the literature with similar lipid profiles observed in large antral follicles. Taken together, the differences in lipid profiles within the follicular fluid are related to the preparation of the dominant follicle for ovulation. Circulating steroids, specifically estradiol, were negatively correlated with phthalic acid and HemeA within the follicular fluid after FSHLow. This negative correlation might be an indicator of the status of the follicle. Increased production of these lipids can lead to a shift in follicular fluid cytokine concentrations, regulating the inflammatory process within the follicle. 

Oocyte quality is impacted by the surrounding cumulus cells, which require lipids and cytokines to drive the proliferation of the cells [74]. Specifically, interferon beta (IFNβ) is linked to reduced estradiol production from granulosa cells and increased cumulus cell expansion during the dominant follicle stage [13,75]. Increased concentrations of IFNβ in FF were observed in response to FSHLow, which can be related to increases in the expansion of cumulus cells to allow for greater oocyte competency. While oocyte quality was not analyzed in this study, the differences in lipid and cytokine profiles in the follicular fluid can serve as an indicator of the follicular environment. Future studies on the relationship of the combination of these lipids and cytokines on oocyte quality and maturation are warranted.

## 5. Conclusions

The current study provides novel information on changes in the expression of lipids and cytokines and their relationship to circulating steroid hormones before and after a low dose of FSH. The FSHLow protocol provides a perspective of changes leading to ovulation and how molecules in circulation and follicular fluid differ during the estrous cycle. The levels of specific lipids and cytokines identified in these studies may serve as markers critical for the ovulatory process and may be used to determine complications associated with anovulation. 

## Figures and Tables

**Table 1:** Pearson correlations between steroid hormones and lipids in plasma at DFA (EL) and after FSH stimulation at FSHLow.

		Plasma			
		DFA		FSHLow	
	Lipid Name	R^2^	*p*-Value	R^2^	*p*-Value
E2	TG 54:6			−0.83	0.02
	TG 58:6			−0.80	0.03
	HemeA			−0.80	0.04
A4	Cholesteryl-11-hydroperoxy-eicosateraenoate			0.87	0.01
	DG 38:0	0.79	0.04		
	TG 49:1	0.79	0.04		
	TG 50:0	0.78	0.05		
	TG 54:6	0.80	0.05		
	TG 58:6	0.85	0.01		
P4	PC 38:2	0.78	0.05		
	3-Deoxyvitamin	0.82	0.02		
	Oleamide	0.87	0.01		
	sn-glycero-3-phosphoserine	0.82	0.02		
	CE 16:0	0.88	0.02		
	C.E.	0.80	0.03		
	HODE			0.84	0.02
	DG 16:0–18:0	0.77	0.05		
	DG 42:6	0.86	0.01		
	TG 52:0	0.77	0.05		
	16:0 SM (d18:1/16:0)	0.87	0.01		
	Diolein	0.83	0.02		
	HemeA			0.83	0.02

**Table 2:** Pearson correlations between steroid hormones and lipids in follicular fluid at DFA or FSHLow.

		Follicular Fluid			
		DFA		FSHLow	
	Lipid Name	R^2^	*p*-Value	R^2^	*p*-Value
E2	1,2-dilinoleoyl sn-glycero-3-phosphocholine	−0.96	0.01		
	Lyso-PC20:4	−0.95	0.02		
	Phthalic acid			−0.79	0.04
	HemeA			−0.87	0.01
A4	PC34:2	−0.94	0.02		
	PC36:0	−0.97	0.004		
	sodium glycochenodeoxycholate			−0.81	0.03
	DG42:6			0.86	0.01
	1-oleoyl-2-hydroxy-sn-glycero-3-phosphocholine	0.90	0.05		
P4	Phthalic acid	0.91	0.04		
	1,2-dilinoleoyl sn-glycero-3-phosphocholine			0.83	0.02

**Table 3:** Pearson correlations between steroid hormones and cytokines in plasma during different stages of the estrous cycle (EL, ML) and after FSH stimulation (FSHLow).

		Plasma					
		EL		ML		FSHLow	
	Cytokine	R^2^	*p*-Value	R^2^	*p*-Value	R^2^	*p*-Value
E2	Decorin					0.81	0.03
	IFNα					0.77	0.05
	IL-10					0.81	0.03
	IL-18					0.79	0.04
	RANTES					0.79	0.04
A4	IL-1αA	0.85	0.01				
	IL1-F5	0.79	0.04				
	RANTES	−0.84	0.02				
	TNFα	0.83	0.02				
	IL-1β			0.82	0.02		
	IL-13			0.85	0.01		
	IL-17αA			0.82	0.01		
	IL-21			0.82	0.02	0.81	0.03
	MIG			0.81	0.03	0.80	0.03
P4	CD40L	0.83	0.02			0.82	0.02
	IL-13			−0.81	0.03		
	IL17αA			−0.80	0.04		
	MIG			−0.82	0.03		

**Table 4:** Pearson correlations between steroid hormones and cytokines in follicular fluid at DFA (EL) and after FSH stimulation (FSHLow).

		Follicular Fluid			
		DFA		FSHLow	
	Cytokine	R^2^	*p*-Value	R^2^	*p*-Value
E2	MIG	−0.82	0.02		
P4	ANGPT1			0.79	0.05
	RANTES	0.84	0.02		

## Data Availability

Data will be made available upon reasonable request to the corresponding author.

## Supplementary Materials

The following supporting information can be downloaded at: https://www.mdpi.com/article/10.3390/ani13203289/s1, Table S1: Complete Plasma Lipid Compounds from EL, ML and FSHLow. Table S2: Complete Follicular Fluid Lipid Compounds from EL, ML and FSHLow.
